# Resveratrol Enhances mRNA and siRNA Lipid Nanoparticles Primary CLL Cell Transfection

**DOI:** 10.3390/pharmaceutics12060520

**Published:** 2020-06-07

**Authors:** Edo Kon, Inbal Hazan-Halevy, Daniel Rosenblum, Niv Cohen, Sushmita Chatterjee, Nuphar Veiga, Pia Raanani, Osnat Bairey, Ohad Benjamini, Arnon Nagler, Dan Peer

**Affiliations:** 1 Laboratory of Precision NanoMedicine, Tel Aviv University, Tel Aviv 69978, Israel; edokon89@gmail.com (E.K.); hinbal@tauex.tau.ac.il (I.H.-H.); danielr0406@gmail.com (D.R.); nivcohenb@gmail.com (N.C.); sushmita.microbio@gmail.com (S.C.); nuphar.veiga@gmail.com (N.V.); 2 School of Molecular Cell Biology and Biotechnology, George S Wise Faculty of Life Sciences, Tel Aviv University, Tel Aviv 69978, Israel; 3 Department of Materials Sciences and Engineering, Iby and Aladar Fleischman Faculty of Engineering, Tel Aviv University, Tel Aviv 69978, Israel; 4 Center for Nanoscience and Nanotechnology, and Tel Aviv University, Tel Aviv 69978, Israel; 5 Cancer Biology Research Center, Tel Aviv University, Tel Aviv 69978, Israel; 6 Davidoff Cancer Center, Beilinson Hospital, Rabin Medical Center Petah Tiqva, Institute of Hematology, Petah Tikva 49100, Israel; Piar@clalit.org.il (P.R.); Osnatb@clalit.org.il (O.B.); 7 Chaim Sheba Medical Center, Tel-Hashomer, Ramat Gan 52621, Israel; Ohad.Benjamini@sheba.health.gov.il (O.B.); a.nagler@sheba.gov.il (A.N.)

**Keywords:** endosomal escape, B lymphocyte, RNA therapies, drug delivery, clinic, caffeic acid, curcumin

## Abstract

Chronic lymphocytic leukemia (CLL) is the most common adult leukemia in Western populations. Therapies such as mRNA and siRNA encapsulated in lipid nanoparticles (LNPs) represent a clinically advanced platform and are utilized for a wide variety of applications. Unfortunately, transfection of RNA into CLL cells remains a formidable challenge and a bottleneck for developing targeted therapies for this disease. Therefore, we aimed to elucidate the barriers to efficient transfection of RNA-encapsulated LNPs into primary CLL cells to advance therapies in the future. To this end, we transfected primary CLL patient samples with mRNA and siRNA payloads encapsulated in an FDA-approved LNP formulation and characterized the transfection. Additionally, we tested the potential of repurposing caffeic acid, curcumin and resveratrol to enhance the transfection of nucleic acids into CLL cells. The results demonstrate that the rapid uptake of LNPs is required for successful transfection. Furthermore, we demonstrate that resveratrol enhances the delivery of both mRNA and siRNA encapsulated in LNPs into primary CLL patient samples, overcoming inter-patient heterogeneity. This study points out the important challenges to consider for efficient RNA therapeutics for CLL patients and advocates the use of resveratrol in combination with RNA lipid nanoparticles to enhance delivery into CLL cells.

## 1. Introduction

Chronic lymphocytic leukemia (CLL) is the most common leukemia, with 15,000 new diagnoses and claiming 5000 lives per year in the United States alone [1,2,3,4]. The median age of CLL patients at diagnosis is above 70 years [1,3]. The disease is characterized by the progressive aggregation of phenotypically mature non-functioning, non-proliferating malignant B lymphocytes arrested in the G0/G1 stage. CLL cells can be found primarily in the peripheral blood, bone marrow, and lymph nodes. They express both B cell markers, such as cluster of differentiation (CD)19, together with non-B cell markers, such as CD5 and CD23 [3,4,5,6]. While asymptomatic CLL manifestations are generally not treated, the majority of cases do require medical intervention [4]. Currently, the standard treatment for CLL is a harsh chemo-immunotherapy regimen of fludarabine (purine analog), cyclophosphamide or chlorambucil (alkylating agents) and rituximab (α-CD20 antibody), also known by its acronym FCR, which is not applicable for all patients [3,7].

RNA-based therapies can be utilized as a versatile therapeutic platform for many diseases. They can silence gene expression, express proteins, edit genes, and serve as specific cancer and viral prophylactic vaccines [8]. Presently, there is much enthusiasm in advancing new RNA-based therapies leading to both in vivo and ex vivo RNA-based therapies [9]. This is propelled by technological advancements in delivery vehicles, which are demanded due to the innate limitations and challenges of RNA therapies. These limitations and challenges include RNA instability, a lack of cellular uptake, minimal endosomal escape, toxicity, and short in vivo circulation time [9]. Lipid nanoparticles (LNPs) are the leading delivery vehicle for RNA therapeutics, with many therapeutics in clinical trials and one FDA-approved LNP-based nanomedicine (ONPATTRO^®^ (Patisiran)) [10,11]. RNA-encapsulating LNPs confer an efficient biological effect, close to 100% payload encapsulation with minimal toxicity [12,13].

Primary B cells are notoriously hard to transfect with nucleic acids. This feat has been accomplished mainly by electroporation, which cannot be recapitulated successfully in vivo for therapeutics and is toxic to cells [5,14]. CLL cells, which are B cells, are small, have minimal cytoplasm volume, and pack a dense nucleus containing aggregated chromatin without nucleoli [3]. To deliver nucleic acids to the cytoplasm of cells, LNPs must both internalize and escape their endosomal compartments before they fuse to lysosomes. This is accomplished by including a pH-dependent ionizable lipid, which facilitates endosomal escape, in the LNPs structure [15,16]. However, studies demonstrate that endosomal escape remains a major bottleneck in the field, and even with ionizable lipids, the endosomal escape of RNA molecules is very low [17,18]. In our study, we aimed to characterize the process of CLL transfection with RNA-LNPs and propose how to overcome the bottlenecks in primary CLL cell transfection to develop future RNA-LNP therapies for this disease.

Polyphenols are a group of biologically active compounds in plant-based foods and are considered the most frequent antioxidants in our diet [19]. Presently, there is an increased interest in repurposing plant-derived polyphenols due to their suggested safety and therapeutic potential, including oncology. Among these, resveratrol, caffeic acid, and curcumin have all been proposed to have anti-cancer activities [19,20,21,22]. In our study, we aimed to determine the potential to repurpose these polyphenols in combination with RNA-LNPs and examine if there is an added benefit. Resveratrol, a well-known plant polyphenol most commonly found in grape seeds and red wine, carries intrinsic antioxidant properties with a plethora of health benefits reported. Among these, resveratrol was reported to have anti-inflammatory, cancer chemo-preventive, anti-aging, neuroprotective, and cardiac-beneficial properties. Regarding carcinogenesis, resveratrol was reported to inhibit cellular events associated with tumor initiation, promotion, progression, and angiogenesis [23,24,25].

In this study, we demonstrate how to effectively transfect primary CLL patient samples, overcoming inter-patient heterogeneity. We delineate the major bottlenecks preventing the translation of RNA-based medicines for this disease. We report that resveratrol enhances RNA-LNP-based cellular manipulations. Importantly, we achieve this with an FDA-approved LNP formulation.

## 2. Materials and Methods

### 2.1. Materials

Cholesterol, distearoyl-sn-glycero-3-phosphocholine (DSPC), and dimyristoyl-rac-glycero-3-methoxypolyethylene glycol (PEG-DMG) were from Avanti lipids (Alabaster, AL, USA). [(6Z,9Z,28Z,31Z)-heptatriaconta-6,9,28,31-tetraen-19-yl] 4-(dimethylamino)butanoate (Dlin-MC3-DMA) (MC3) was synthesized in-house. Quant-it Ribogreen was from Thermo Fisher scientific (Waltham, MA, USA). Cells were fractionated using Ficoll-Paque™ PLUS from GE Healthcare (Chicago, IL, USA). All labeled and non-labeled siRNA sequences were from IDT technologies (Coralville, IA, USA). Custom mRNA-Luc was from Trilink (San Diego, CA, USA). Annexin-V and PI are products of Biolegend (San Diego, CA, USA) and Sigma-Aldrich (St Louis, MO, USA), respectively. Invitrogen™ Quant-iT™ RiboGreen™ RNA Assay Kit was from Thermo Fisher Scientific (Waltham, MA, USA). Gibco RPMI media was fromThermo Fisher Scientific (Waltham, MA, USA). APC α-human CD44 was from Biolegened (San Diego, CA, USA). Resveratrol, caffeic acid, curcumin, and chloroquine were from Sigma-Aldrich (St Louis, MO, USA). For luciferase assays, Promega Luciferase assay systems kits were used (Madison, WI, USA). RNA was extracted from cells with a GeneJET RNA Purification Kit from Thermo Scientific™ (Waltham, MA, USA). A qScript cDNA Synthesis Kit from Quantabio was used to synthesize cDNA (Beverly Hills, CA, USA). RT-PCR reactions were carried out with Fast SYBR™ Green Master Mix from Applied Biosystems™/Thermo Scientific™ (Waltham, MA, USA).

### 2.2. LNP Preparation and Characterization

Dlin-MC3-DMA (MC3), cholesterol, DSPC, and PEG-DMG were mixed at a molar ratio of 50:38:10.5:1.5 with absolute ethanol in a tube. Acetic acid buffer [25 mM] and citric acid buffer [50 mM] were used to suspend siRNA and mRNA, respectively. For the uptake experiments, Cy5-labeled negative control siRNA (NC-Cy5) was used at 30% of total RNA amount. To create LNPs, a dual syringe pump was used to transport the two solutions through the NanoAssembler™ micromixer from Precision NanoSystem (Vancouver, British Columbia, Canada) at a total flow rate of 12 mL/min. The particles were then transferred into dialysis overnight against PBS. Particles in PBS were analyzed for size and uniformity by dynamic light scattering (DLS). Zeta potential was determined using the Malvern™ zeta-sizer (Malvern, Worcesrershire, UK). RNA encapsulation in LNPs was calculated according to Quant-iT™ RiboGreen™ RNA Assay Kit (Thermo Fisher, Waltham, MA, USA), by calculating the percentage encapsulation at 100% - (RNA-LNPs/RNA-LNPs with triton).

### 2.3. Transfection Protocol

Primary patient samples were thawed, cells were plated without serum, and transfection was performed by adding 4 µg/mL or 2.5 µg/mL of siRNA and mRNA encapsulated in LNPs, respectively. Cells were grown for 1 h at 37 °C and 5% CO_2_ in RPMI media supplemented with penicillin–streptomycin solution, L-glutamine, and sodium pyruvate. After 1 h, 5–40 µM resveratrol was added directly to the wells (DMSO concentration < 0.05% at all times). Cells were incubated for another 2 h at 37 °C and 5% CO_2_ followed by supplementation of FCS to a total of 10% of the well volume.

### 2.4. Separation of CLL Cells from Patient Samples

Whole blood was obtained from patients with CLL prior to treatment at the Sheba Medical Center Clinics at Tel-Hashomer Hospital and at the Rabin Medical Center-Beilinson Hospital, after obtaining institutional review board-approved informed consent. CLL blood cells were fractionated using Ficoll-Paque™ PLUS. Peripheral blood mononuclear cells were extracted from the gradient and were frozen in a 10% DMSO and 90% FCS freezing solution. Product purity was determined by co-staining with CD5- and CD19-labeled antibodies (Figure S1). A table containing characteristics of patient samples can be found in supplementary section (Table S1).

### 2.5. LNP Uptake Experiments

CLL patient samples were suspended in FACS tubes at a density of 10^6^ cells/250 µL media with or without serum. Cells were transfected by coincubation with 4 µg/mL of NC-Cy5-LNPs at 37 °C. At described timepoints, tubes were washed twice with PBS and fluorescence was determined by flow cytometry.

### 2.6. Confocal Microscopy Analysis

Cells were plated at a density of 4 × 10^6^ cells/well in 12-well plates covered with coverslips. Cells were transfected with 4 µg/mL of NC-Cy5-LNPs, and the transfection method was carried out as described. For Hoechst staining, plates were centrifuged and wells were washed with PBS and stained with 2.5 µg/mL Hoechst live staining for 30 min at 37 °C and 5% CO_2_. Next, plates were centrifuged again, washed with PBS, and fixated with 4% paraformaldehyde for 30 min at room temperature. After 30 min, the paraformaldehyde was aspirated and wells were blocked with 3% BSA for 1 h at room temperature. Cell membranes were stained with APC-αhuman CD44 (diluted 1:100 in 3% BSA) for 30 min at 4 °C in dark. Wells were washed twice with PBS and coverslips were mounted on slides and imaged by confocal microscopy.

### 2.7. Luciferase Expression

Cells were plated at a density of 10^6^ cells/well in 48-well plates, transfected with 2.5 µg/mL of luciferase mRNA encapsulated in LNPs. Expression was determined 24 h post-transfection according to the Promega kit protocol, relative luminescence units were determined by a Veritas™ microplate luminometer. Toxicity was determined 48h post transfection by PI ANNEXIN-V, as described previously.

### 2.8. Mcl-1, CD44, STAT3 Silencing, and Cell Viability Assessment

Cells were plated at a density of 4 × 10^6^ cells/well in 12-well plates, transfected with 4 µg/mL of siRNA targeted against Mcl-1, CD44, or STAT3 encapsulated in LNPs by directly adding LNPs to the media. Viability was assessed 48 h post transfection by PI ANNEXIN-V staining. Briefly, cells were collected, washed twice with PBS, and followed by a 15-min incubation at room temperature with 100µl of annexin binding buffer containing propidium iodide (1:40) and Annexin–APC (1:20). Cell death percentage was determined by flow cytometry. The siRNA sequences employed in study:

siMcl-1 sense: CCCGCCGAAUUCAUUAAUUUACUGT,
anti-sense: ACAGUAAAUUAAUGAAUUCGGCGGGUA;
siCD44 sense: GGCGCAGAUCGAUUUGAAUAUAACC,
anti-sense: GGUUAUAUUCAAAUCGAUCUGCGCCCAG;
siSTAT3 sense: CAGCAACACUCUUCAGUACAUAAUA,
anti-sense: UAUUAUGUACUGAAGAGUGUUGCUGGA;
siNC5 sense: CAUAUUGCGCGUAUAGUCGCGUUAG,
anti-sense: UGGUAUAACGCGCAUAUCAGCGAAUC.

### 2.9. RT-PCR Experiments

Total RNA was extracted from CLL cells with the Thermo Scientific GeneJET RNA Purification Kit, according to the manufacturer’s protocol. The cDNA was synthesized from an initial amount of 500 ng of total RNA extracted with a qScript cDNA Synthesis Kit, according to the manufacturer’s protocol. The cDNA was diluted 1:3 in nuclease-free water and RT-PCR was carried out. In all experiments, expression was normalized to subunit A of eukaryotic initiation factor 3 (eIF3A). Primer sequences employed in the study can be found in supplementary section (Table S2).

## 3. Results

### 3.1. Physicochemical and Structural Characterization of LNPs

**Figure 1:**
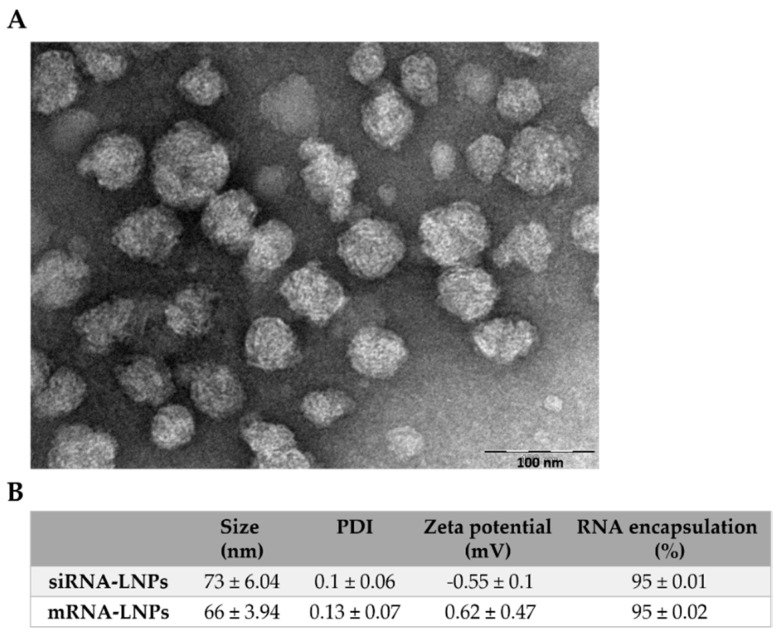
Physicochemical and structural characterization of lipid nanoparticles (LNPs). (**A**) A representative (transmission electron microscopy) TEM image of RNA-encapsulating LNPs. (**B**) Table summarizing LNP physicochemical aspects.

To transfect CLL cells with both siRNA and mRNA, we employed an FDA-approved lipid formulation of Dlin-MC3-DMA (MC3), cholesterol, DSPC, and PEG-DMG; mixed at a molar ratio of 50:38:10.5:1.5, respectively. This formulation is considered the current gold standard formulation for RNA-LNP therapy. It is approved as the siRNA LNP treatment ONPATTRO^®^ (patisiran), an RNAi therapeutic for hereditary transthyretin amyloidosis (hATTR [11,26,27,28]. The LNPs were prepared by microfluidic mixing with the NanoAssemblerTM micromixer. This enables both high inter-batch reproducibility, as well as the intra-batch uniformity of LNPs. The hydrodynamic diameter of these LNPs was measured to be 73 ± 6.04 nm and 66 ± 3.94 nm for siRNA- and mRNA-encapsulating LNPs, respectively, with close to 100% encapsulation efficiency. Consistently uniform LNPs were formed with a polydispersity index (PDI) of <0.2, with a close to neutral zeta potential (Figure 1B).

### 3.2. Serum Depletion Results in Rapid LNP Uptake into CLL Cells which Enhances mRNA-LNPs Transfection

**Figure 2:**
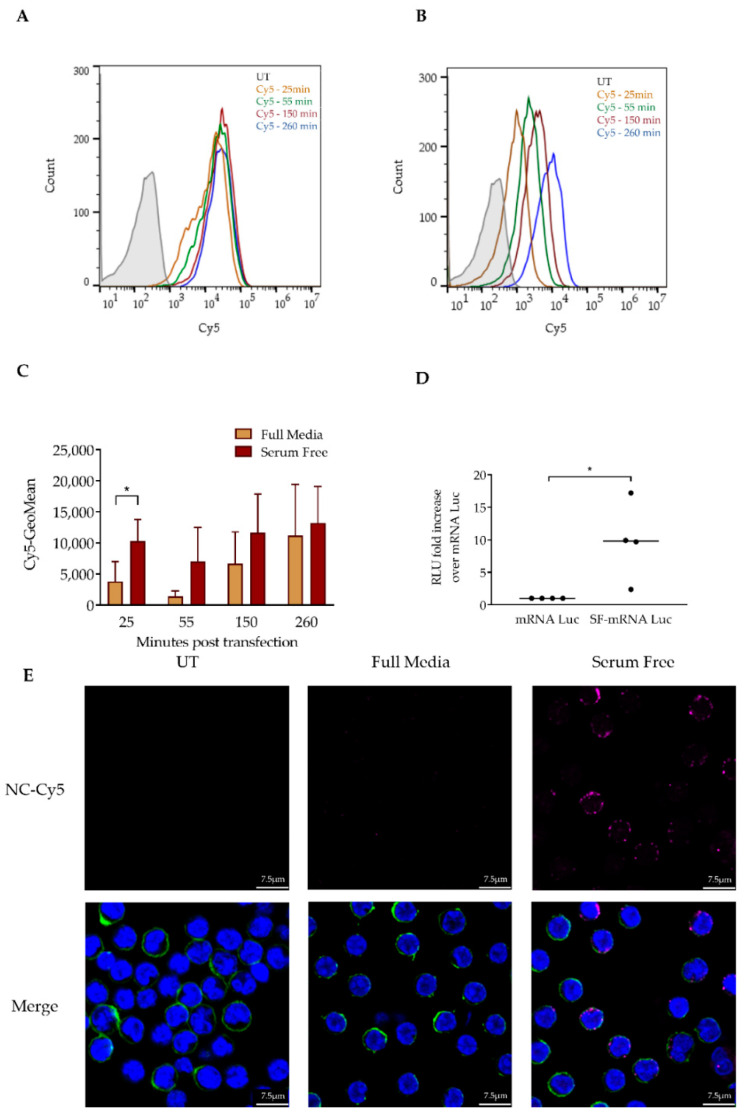
Serum depletion results in rapid LNP uptake into chronic lymphocytic leukemia (CLL) cells. (**A**) CLL patient samples were co-incubated with 4 µg/mL of NC-Cy5-LNPs in serum-free media (SF). Fluorescence was determined 25, 55, 150 and 260 min post transfection by flow cytometry. (**B**) CLL samples were transfected and co-incubated with 4 µg/mL of NC-Cy5-LNPs in media containing 10% fetal calf serum (FCS). Fluorescence was determined 25, 55, 150 and 260 min post transfection by flow cytometry. (**C**) Geometric mean of NC-Cy5-LNPs signal determined at sequential timepoints (*n* = 3). (**D**) Comparison of fold change in the expression of Luciferase mRNA encapsulated in LNPs (mRNA-Luc). CLL patient samples (*n* = 4) were transfected in 10% FCS or serum-free media with 2.5 µg/mL mRNA-Luc. After 3 h, FCS was replenished to a total of 10% of well volume in serum-free conditions. Luciferase expression was determined 24 h post transfection by a luminometer; measured in relative luminescence units (RLU). (**E**) Images of cells transfected with 4 µg/mL NC-Cy5-LNPs (red) 25 min post transfection in full or serum-free media. Cells were stained with Hoechst (blue) and CD44 membrane staining (green). Nucleus and membrane staining has been removed from top row images to better visualize LNP uptake. Imaged by confocal microscopy. For all experiments: (*) *p* < 0.05 (two-sided Student’s *t*-test).

First, we aimed to gain insight into the process of CLL cell transfection by RNA-LNPs. The efficient transfection of primary CLL cells with the FDA-approved LNP formulation remains a formidable challenge. Therefore, we aimed to understand the factors that impede this process. First, we characterized LNP uptake into CLL cells and interrogated how serum proteins affect LNP uptake into these cells. To test this, we co-incubated primary CLL cells with LNPs encapsulating a Cy5-labeled negative control (NC) siRNA (NC-Cy5-LNPs) and measured the fluorescence of the CLL cells by flow cytometry at sequential time points. The incubation of NC-Cy5-LNPs with cells in serum-free media resulted in a rapid increase in LNP uptake compared to full media conditions (Figure 2A,B). The difference in uptake was most significant at the 25 min timepoint and gradually evened out (Figure 2C). The rapid uptake of NC-Cy5-LNPs in serum-free conditions was corroborated by confocal microscopy imaging (Figure 2E). Next, we aimed to understand how this rapid uptake affects LNP transfection. We incubated primary CLL patient samples with mRNA luciferase-encapsulating LNPs (mRNA-Luc) and measured luminescence 24 h post transfection. Interestingly, while the eventual uptake was nonsignificant, the rapid uptake did result in a significant increase in mRNA-Luc transfection. This was witnessed in all samples tested, overcoming interpatient heterogeneity associated with studies executed with primary cells (Figure 2D). From this, we conclude that the rate of LNP uptake is crucial for successful transfection in primary CLL cells.

### 3.3. Resveratrol Enhances mRNA LNP Transfection into Primary CLL Cells

**Figure 3:**
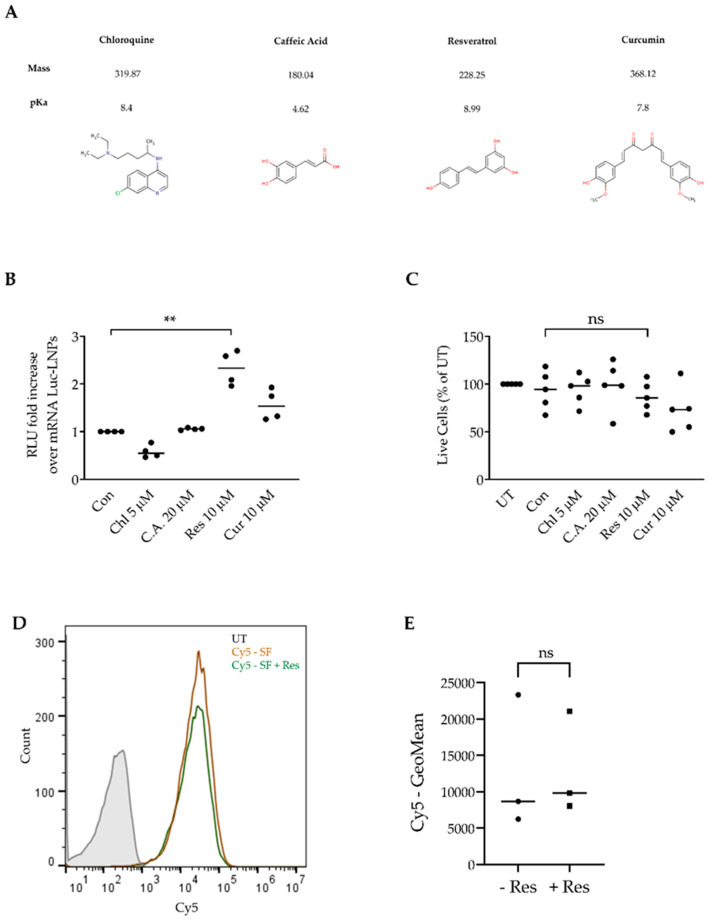
Resveratrol enhances mRNA-LNP transfection into primary CLL cells. (**A**) Chemical structures and properties of chloroquine, caffeic acid, resveratrol and curcumin. Taken from the Chemical Entities of Biological Interest (ChEBI) database. (**B**) Comparison of fold change in the expression of Luc-mRNA. CLL patient samples (*n* = 4) were transfected in serum-free media with 2.5 µg/mL of mRNA-luciferase encapsulated in LNPs (mRNA-Luc). After 1 h, resveratrol (Res), caffeic acid (C.A), curcumin (Cur), and chloroquine (Chl) were added at a concentration of 10 µM, 20 µM, 10 µM, and 5 µM, respectively. After 3 h, FCS was replenished to a total of 10% of well volume. Luciferase expression was determined 24 h post transfection by a luminometer; measured in relative luminescence units (RLU). (**C**) Viability was measured 48 h post transfection by propidium iodide (PI) and annexin-V staining, as a percentage of the untreated sample; determined by flow cytometry. (**D**) Resveratrol’s effect on LNP uptake (representative image). Primary CLL cells were incubated in serum-free conditions in 37 °C with 4 µg/mL NC-Cy5-LNPs for 1 h. After 1 h, resveratrol was added. After 3 h, Cy5 fluorescence was measured by flow cytometry. (**E**) Comparison of geometric mean of Cy5 fluorescence after resveratrol addition. For all experiments: (*) *p* < 0.05, (**) *p* < 0.01 (two-sided Student’s *t*-test).

Next, we screened three different plant-based polyphenols for the ability to enhance mRNA-Luc-LNP transfection. Plant-based polyphenols are reported to have innate anti-cancer properties and are suggested for the supplementation of cancer therapies. Therefore, we aimed to determine the ability to harness three safe and well-known polyphenols with RNA-LNP transfection. We chose to compare resveratrol, caffeic acid, and curcumin (Figure 3A). All of these plant-based polyphenols were reported to have anti-cancer properties [19,20,21,22]. To evaluate the polyphenols’ capabilities, we co-incubated mRNA-Luc-LNPs with primary CLL patient samples in serum-free media conditions for 1 h. After 1 h, 5–40 µM of resveratrol, caffeic acid, curcumin, and chloroquine were added to the wells. Chloroquine, an antimalarial that is known to affect the endolysosomal system, serves as a positive endosomal escape control in many studies due to increasing endosomal escape, was utilized as a positive control for our screen [29,30,31]. The optimal concentration of each agent was determined by the maximum enhancement of mRNA-Luc transfection measured after 24 h and minimal toxicity measured after 48 h (Figure S2). The screen, performed on different patient samples, demonstrates that, of the polyphenols tested, both curcumin and resveratrol enhance transfection at low micro-molar concentrations (Figure 3B). Of these, resveratrol at 10 µM demonstrated the best potential for augmenting mRNA-Luc transfection into CLL cells with no toxicity (Figure 3C). Surprisingly, chloroquine treatment did not augment mRNA-Luc transfection (Figure 3B). Since we added resveratrol into our transfection protocol 1 h post transfection, after the LNP saturation of cells in serum-free conditions, we did not expect resveratrol to cause increased LNP uptake. To validate this, we co-incubated NC-Cy5-LNPs in serum-free media, added resveratrol, and determined whether uptake was enhanced. As expected, the addition of resveratrol did not affect LNP uptake (Figure 3D,E). This suggests that the resveratrol enhancement of transfection is not due to increased LNP uptake.

### 3.4. Resveratrol Enhances siRNA LNP Transfection into Primary CLL Cells

**Figure 4:**
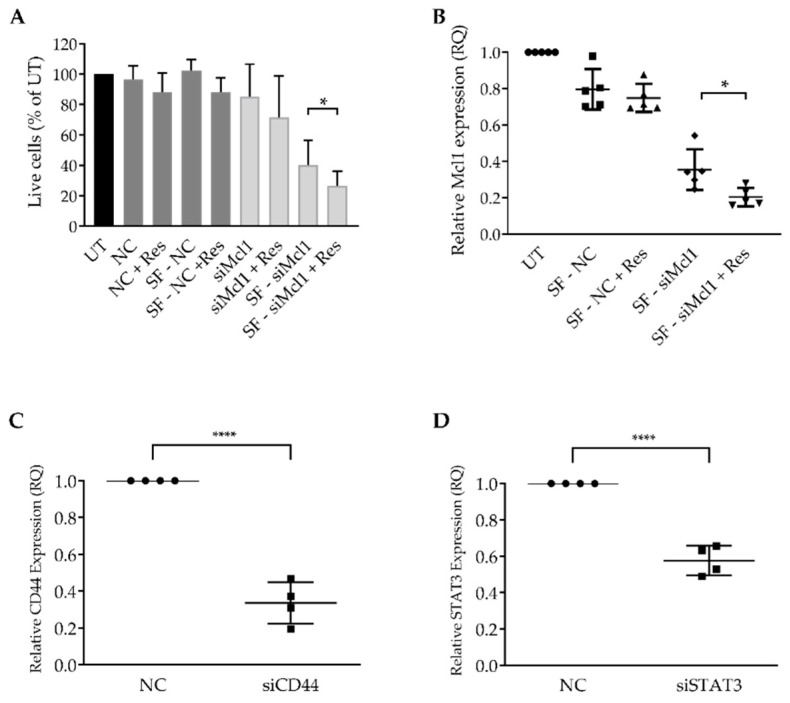
Transfecting primary CLL cells with siRNA targeting Mcl-1, STAT3, and CD44. (**A**) Patient samples were transfected in full or serum-free (SF) media, with 4 µg/mL of Mcl1 targeting or non-targeting control (NC) siRNA encapsulated in LNP. After 1 h, 30µM resveratrol (Res) was administered where noted. After 3 h, fetal calf serum (FCS) was replenished to a total of 10% in each well. Cell viability was measured 48 h post transfection by propidium iodide (PI) and annexin-V staining, as a percentage of the untreated sample; determined by flow cytometry. (**B**) Mcl1 gene expression levels were determined by RT-PCR, 24 h post transfection. (**C**,**D**) Patient samples were transfected in serum-free media with 4µg/mL of CD44, STAT3, or NC-siRNA encapsulated in LNPs. After 1 h, 30 µM resveratrol was added to all wells. After 3 h, FCS was replenished to a total of 10% of the total well volume. CD44 and STAT3 gene expression levels were determined by RT-PCR, 48 h post transfection. (*) *p* < 0.05, (**) *p* < 0.01, (***) *p* < 0.001), (****) *p* < 0.0001 (two-sided Student’s *t*-test).

Next, we aimed to test if resveratrol augments siRNA-mediated gene silencing. First, we co-incubated primary CLL patient cells with Mcl-1-targeting siRNA encapsulated in LNPs (Mcl-1-siRNA). Mcl-1, a member of the Bcl-2 family, is over-expressed in CLL, as well as in other leukemias. Mcl-1 enhances CLL cell survival by inhibiting apoptosis and is associated with drug resistance [32,33,34]. Therefore, to evaluate siRNA transfection, we executed a phenotypic screen and determined cell viability 48 h post transfection (Figure 4A). To confirm that this phenotype is a result of Mcl-1 gene knockdown, we also measured gene expression (Figure 4B). The results demonstrate that the transfection enhancement by adding resveratrol with minimal to no toxicity was brought on by the process itself (Figure 4A,B). We could notice Mcl-1 gene silencing as early as 24 h post transfection and determined that, while there is an added benefit to transfection in serum-free media, administering resveratrol significantly enhances the transfection efficiency (Figure 4B).

Next, to demonstrate that this method can be applied to several genes, we chose to transfect primary CLL cells with LNPs encapsulating siRNA targeting CD44 and signal transducer and activator of transcription 3 (STAT3). STAT3 is important for CLL cellular growth, survival, and has a role in CLL immunosuppression and drug resistance [35,36,37]. CD44 is a well-known cancer stem cell-associated marker which has also been reported to participate in CLL cell survival [38,39]. To this end, we transfected primary CLL cells with si-CD44 and si-STAT3 and determined CD44 and STAT3 gene expression levels (Figure 4C,D). These results demonstrate that resveratrol enhances not only mRNA, but also siRNA-LNP transfection.

## 4. Discussion

Herein, we interrogated the process of LNP uptake into primary CLL patient cells. We demonstrate how rapid the uptake of RNA-LNPs can lead to the efficient transfection of these cells. CLL cells, similar to most leukocytes, are notoriously hard to transfect with any tool in a non-toxic fashion. Additionally, while primary CLL cells survive for long periods in vivo, they undergo rapid apoptosis ex vivo, which complicates the ability to study primary patient samples [5,40]. Furthermore, we demonstrate the capability of repurposing resveratrol as an RNA-LNP transfection enhancer for both mRNA and siRNA payloads, hinting at a possible shared mechanism. In our study, we employed an FDA-approved LNP formulation, which is considered the current gold standard formulation for RNA-LNP therapy. It is approved as an siRNA-LNP therapeutic for hereditary transthyretin amyloidosis (hATTR); ONPATTRO^®^ (patisiran), approved in 2018 [11,26,27,28]. Furthermore, LNPs demonstrate acceptable toxicities and are currently assessed in clinical trials in the oncology field, as well as for prophylactic viral vaccines for many infections, including Zika (NCT04064905) and the novel SARS-CoV-2 (NCT04283461) [9,41].

Several interesting points remain to be elucidated following this study. First, the relationship between the short serum depletion and the dramatic increase in LNP uptake, which can be a result of either cellular changes or alterations in the LNP properties. Serum depletion can be related to metabolic stress and has been reported to induce G1/G0 cell cycle arrest [42,43]. G1/G0 cell cycle arrest leads to increased endocytosis, the main pathway of LNP uptake into cells. Yet, CLL cells are already permanently arrested in the G1/G0 stage [5]. Further complicating the matter, several previous reports demonstrate that the absence of serum from media inhibits efficient transfection with LNPs. The relationship between serum presence and transfection efficacy is tricky to predict and is proposed to be related to the LNP formulation and cells targeted [44,45]. Regarding LNPs’ involvement, serum proteins can coat LNPs and alter their physical properties; this phenomenon is known as a “protein corona” [17]. While it is known, it is very hard to predict which protein corona enrichment pattern will emerge, since it depends on many factors, such as the formulation and type of PEG-lipids used [44]. In our study, we demonstrate that an important factor is the speed with which the primary cells are loaded with LNPs. It would be interesting to further interrogate and suggest a mechanism for this phenomenon. Furthermore, in future studies, we will attempt to address what is driving this rapid uptake to try to enhance this process to develop future targeted RNA-LNP-based therapeutics for CLL patients. In the study, we point out the importance of the kinetics of the uptake and demonstrate that the rapid uptake of LNPs is key to efficient transfection.

In our study, we have screened three plant-based polyphenols’ ability to enhance RNA-LNP transfections. Resveratrol, caffeic acid, and curcumin have all been reported to have a myriad of therapeutic properties and are suggested to be quite safe for repurposing [19,20,21,22]. In our screen, we have determined that resveratrol enhances both mRNA- and siRNA-LNP transfections. Since it assists both processes, we suspect that it enhances one of the stages shared by both mRNA and siRNA delivery, such as endosomal escape. Unfortunately, endosomal escape is extremely difficult to quantify in any cell, let alone primary leukocytes, which are small and have a minimal cytoplasmic volume. Furthermore, the process of the endosomal escape of RNA-LNPs is complex and is still being elucidated. Factors such as the molar ratios of ionizable lipids to mRNA nucleotides can modulate the efficiency of RNA-LNPs’ endosomal escape efficiency, intracellular fate, and packaging into extracellular vesicles (EVs). Recently, it was reported that after transfection, LNP components (mRNA and ionizable lipids) are partly incoroporated into exocytosed EVs comprising the encapsulated mRNA and ionizable lipids that can be re-used to efficiently deliver nucleic acids into cells [46].

Regarding resveratrol, several studies have accredited the molecule with cytotoxic and anti-leukemic properties, as well as a role in enhancing the current activity of chemotherapies [47,48,49,50]. In CLL, this was attributed to a tumor-specific increase in apoptosis, associated with a decreased BCL2/BAX ratio [48]. In this study, we have optimized the concentration of resveratrol to be repurposed as a non-toxic transfection enhancer. In the future, it will be very interesting to assess and devise a strategy to combine RNA-LNP therapies with resveratrol supplementation, which can be harnessed for both ex vivo, as well as in vivo, targeted delivery schemes. This will include a therapy scheme involving the co-administration of resveratrol at optimal concentrations, together with RNA-LNPs. Interestingly, it has been suggested that resveratrol could be utilized as an ex vivo therapy to eliminate malignant cells (leukemia purging) during the process of autologous hematopoietic stem-cell transplantation (auto-HSCT) in leukemia patients, due to its proposed innate ability to kill cancer cells specifically [51,52]. Eventually, the optimal in vivo therapy scheme we envision will involve resveratrol, administered together with RNA-LNPs, targeted against CLL cells. We have previously demonstrated a proof of concept of targeted RNA-LNPs for cancer therapeutics, including B cell malignancies [53,54].

## 5. Conclusions

In this study, we report that the rapid uptake of LNPs is key to the successful RNA-LNP manipulation of CLL cells and that the presence of serum slows down this effect. Importantly, we report the potential to harness resveratrol to enhance both mRNA- and siRNA-LNP-based manipulation of primary CLL patient cells.

## Supplementary Materials

The following are available online at https://www.mdpi.com/1999-4923/12/6/520/s1. Table S1: Table with patient characteristics. Table S2: Primers employed in study for RT-PCR reactions. Figure S1: Representative graph of CD5^+^/CD19^+^ patient sample staining. Determined by flow cytometry. Figure S2: Comparison of fold change in expression of Luc mRNA.
