# Supplementary material for: Resveratrol Enhances mRNA and siRNA Lipid Nanoparticles Primary CLL Cell Transfection
Source: Pharmaceutics. 2020 Jun 7;12(6):520. doi: 10.3390/pharmaceutics12060520 (PMC7355647; doi:10.3390/pharmaceutics12060520)
Supplement: Supplementary file 1 [file pharmaceutics-12-00520-s001.pdf]

pharmaceutics

Supplementary Materials: Resveratrol Enhances mRNA and siRNA Lipid Nanoparticles Primary CLL Cell Transfection

Edo Kon, Inbal Hazan-Halevy, Daniel Rosenblum, Niv Cohen, Sushmita Chatterjee, Nuphar Veiga, Pia Raanani, Osnat Bairey , Ohad Benjamini, Arnon Nagler and Dan Peer

Table S1. Table with patient characteristics.

Patient sample	Sex	Age Y	WBCs (103/μl)	Lymphocytes (%)	Rai stage	Previous treatment
**1**	F	68	122500	95	II	None
**2**	M	62	14300	94	II	None
**3**	F	75	388100	90	III	None
**4**	F	75	202700	87	II	Leukeran fludarabine cyclophosphamide rituximab
**5**	M	78	18090	81	I	None
**6**	M	82	28500	75	II	Gammaglobulins
**7**	M	64	280000	92	II	None
**8**	M	64	180200	99	III	FCR
**9**	M	76	163000	96	II	None
**10**	F	87	202700	95	0	None
**11**	F	90	56900	82	0	None

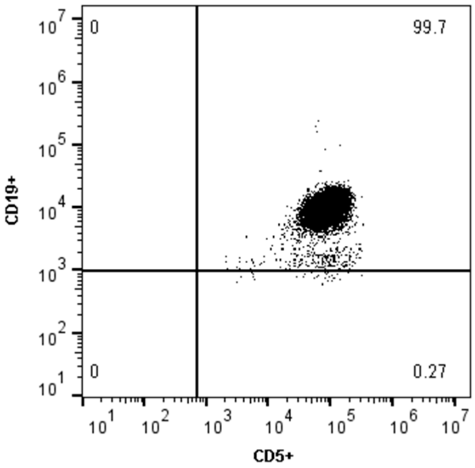

Figure S1. Representative graph of CD5⁺/CD19⁺ patient sample staining. Determined by flow cytometry.

Table S2. Primers employed in study for RT-PCR reactions.

Primer Name	Sequence (5' to 3')
Mcl-1 FWD	AGCTGCATCGAACCATTAGCA
Mcl-1 REV	AACTCCACAAACCCATCCCA
CD44 FWD	GACACATATTGCTTCAATGCTTCAGC
CD44 REV	GATGCCAAGATGATCAGCCATTCTGGATT
STAT3 FWD	CCGAGCCAATTGTGATGCTT
STAT3 REV	TGAGGCCTTGGTGATACACCT
E13A FWD	TCCAGAGAGCCAGTCCATGC
E13A REV	CCTGCCACAATTCCATGCT

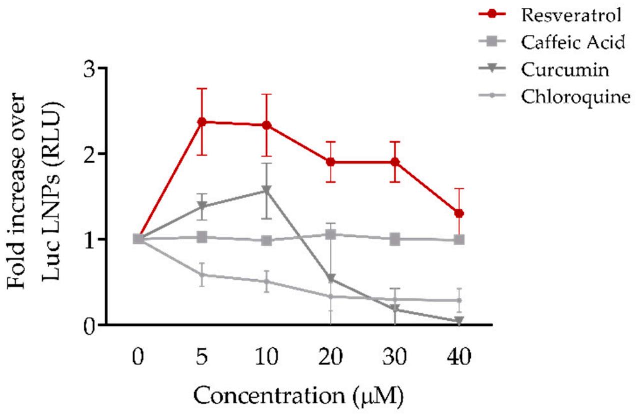

Figure S2. Comparison of fold change in expression of Luc mRNA. CLL patient samples (n=4) were transfected in serum-free media with 2.5 μg/mL of mRNA Luciferase encapsulated in LNPs (mRNA Luc). After 1 h, Resveratrol, Caffeic acid, Curcumin, and Chloroquine were added at a concentration of 5–40 μM. After 3 h, FCS was replenished to a total of 10% of well volume. 24-hour post-transfection, Luciferase expression was determined by Luminometer; measured in relative luminescence units (RLU).

© 2020 by the authors. Licensee MDPI, Basel, Switzerland. This article is an open access article distributed under the terms and conditions of the Creative Commons Attribution (CC BY) license (http://creativecommons.org/licenses/by/4.0/).
